# A Hybrid Leak Localization Approach Using Acoustic Emission for Industrial Pipelines

**DOI:** 10.3390/s22103963

**Published:** 2022-05-23

**Authors:** Yangde Gao, Farzin Piltan, Jong-Myon Kim

**Affiliations:** Department of Electrical, Electronics and Computer Engineering, University of Ulsan, Ulsan 44610, Korea; gaoyangdephd@gmail.com (Y.G.); piltanfarzin@gmail.com (F.P.)

**Keywords:** acoustic emissions, industrial pipelines, minimum entropy deconvolution, damping frequency energy

## Abstract

Acoustic emission techniques are widely used to monitor industrial pipelines. Intelligent methods using acoustic emission signals can analyze acoustic waves and provide important information for leak detection and localization. To address safety and protect the operation of industrial pipelines, a novel hybrid approach based on acoustic emission signals is proposed to achieve reliable leak localization. The proposed method employs minimum entropy deconvolution using the maximization kurtosis norm of acoustic emission signals to remove noise and identify important feature signals. In addition, the damping frequency energy based on the dynamic differential equation with damping term is designed to extract important energy information, and a smooth envelope for the feature signals over time is generated. The zero crossing tracks the arrival time via the envelope changes and identifies the time difference of the acoustic waves from the two channels, each of which is installed at the end of a pipeline. Finally, the time data are combined with the velocity data to localize the leak. The proposed approach has better performance than the existing generalized cross-correlation and empirical mode decomposition combined with the generalized cross-correlation methods, providing proper leak localization in the industrial pipeline.

## 1. Introduction

Industrial pipelines play an important role in the transport of water, oil, and gas. However, long exposures to extremely harsh environments can cause pipeline corrosion, leaks, and even cracks that can lead to environmental pollution and economic loss. If intelligent methods can quickly localize a leak, it can be repaired to reduce loss. Thus, measurement and protection of industrial pipelines are becoming more important [1,2].

Pipelines are easily damaged by load, cracks, and damping during operation. Intelligent methods can detect and analyze various information on the pipeline and are very important. The intelligent methods used in pipelines are typically based on acoustic emission (AE) signals [3,4]. A leak in a pipeline is an AE signal source reflecting negative pressure waves, which can be used to measure industrial pipelines in a real-time transient model using intelligent methods. A received acoustic wave that has traveled through a pipeline can be combined with data regarding mechanisms of leak generation and propagation and applied to intelligent methods for leak localization. A technique is proposed in this study to localize a leak in real time. Leakage noise is caused by the flow of fluid out of the pipeline, which propagates through the fluid inside the pipeline. According to this fundamental principle, acoustic waves propagate in the fluid when a leak occurs in the pipeline; therefore, sensors installed at both ends of the pipeline can collect such data signals. Intelligent methods can analyze these signals and localize the leak based on the distance difference between the signal source and detection point. Specifically, the time difference of arrival (TDOA) algorithm technology is widely used to localize the leak in a pipeline for effective monitoring [5]. The cross correlation (CC) technique is also used for locating leaks in pipelines; depending on the software, a system based on CC performs environmental experiments for AE, taking advantage of the similarity between the channels of data from two sensors installed on the pipeline. This CC technique is also combined with a filtering method to remove background noise; according to the time of the event, a cross correlation function (CCF) can analyze the unit of time correlation and compute the exact leak location using the acoustic wave propagation [6,7,8,9]. However, only a CC method will be precise enough for time delay estimation. TDOA techniques combined with CC can improve the robustness to better than that of the time-of-flight method. Although based on AE wave propagation, the arrival time difference should be relative to the wave velocity that influences the source localization. However, detection of the arrival time is very difficult using the wave amplitude with CCF, which depends on the wave propagation environment, such as the diffraction, divergence, and inner fluent disturbance place, which can decrease the performance of the pipeline localization. A generalized cross correlation (GCC) method, which also uses the cross power spectral density function to change the superior accuracy of the TDOA estimator, was proposed to improve the quality of CCF [10,11,12]. However, various environments and internal factors influence the quality of GCC, hindering prediction accuracy. Therefore, AE sensors installed on the surface of pipelines should be considered for collection of AE waves including noise signals and feature signals. If this noise cannot be removed from mixed signals, it will influence feature analysis performance for intelligent diagnosis. There are various noise-filtering methods. The wavelet transform (WT) method uses the wavelet base to decompose time signals. However, with a wavelet base, WT has no self-adaptability at different scales, and empirical mode decomposition (EMD) can separate mixed signals into several intrinsic mode function (IMF) components and identify feature signals [13,14]. However, there are still problems with this method [15,16]. Though the local mean decomposition (LMD) method can apply the local average and envelope estimation functions to signals, it is limited by the endpoint effect [13,17]. A filtering method can sometimes eliminate noises that are not correlated with the feature leak signals and improve localization accuracy; however, these methods still have intrinsic drawbacks and external factors that limit their use in pipelines. Because of the complex structure containing a variety of noises from the AE signals, the minimum entropy deconvolution (MED) method was applied in this paper to use the kurtosis index to clean raw signals and achieve good results [18,19]. AE signals can be processed by the MED method with time-domain blind de-convolution to extract meaningful information from mixed signals, which are then used for analysis with the feature time difference.

According to TDOA, after the filtering method collects data and obtains intrinsic feature signals, the frequency analysis method is used to extract the energy features from the seismic signals to identify the arrival time difference and calculate the leak location [20,21,22]. The aim of this method is to make sure the time delay and waves attenuate depending on distance and frequency. Additionally, the frequency changes with signal propagation from the beginning of the wave; therefore, the above computational energy methods still have some non-adaptive drawbacks to feature signals for industrial pipelines in complex environments that need to be improved for leak localization. In some papers, the damping term in the dynamic differential equation is used to describe the damping energy under complex geometry, and these architectures also produce some new research ideas that are very useful for pipeline localization in this paper [23,24].

To address the noise influence and adaptive estimate problem for feature impulse signals in an industrial pipeline, a new hybrid approach based on the MED method and damping frequency energy through the damping term into the dynamic differential equation was designed to remove noise, detect arrival time, and compute leak localization accurately for industrial pipelines. In this approach, the damping frequency energy is more adaptive for feature impulse signals. Additionally, the damping frequency energy is linked to MED, which also addresses the noise influence and is very helpful for detecting the leak position. The approach architecture of this method is described as follows. In Section 2, the MED method is described in detail and applied to remove the noise from the AE source waves. In Section 3, the damping frequency energy estimator through damping term into the dynamic differential equation and cross-zero are designed to detect the time difference of the two channels and localize the leak. Section 4 compares existing methods and outlines the experiments, demonstrating that the proposed approach accurately localizes the leak and has better performance than existing methods for industrial pipelines.

## 2. Background of Minimum Entropy Deconvolution

The MED takes advantage of the maximization kurtosis norm to filter noise and extract periodic impulse signals from multiple components in acoustic data. An example is designed to describe the function of MED as

(1) $$\vec{x}=\left[\begin{array}{c} x_{1}\\ x_{2}\\ \begin{array}{c} \vdots \\ x_{n} \end{array} \end{array}\right],\;\vec{d}=\left[\begin{array}{c} d_{1}\\ d_{2}\\ \begin{array}{c} \vdots \\ d_{n} \end{array} \end{array}\right],\;\vec{x}=\vec{d}+\vec{e}$$

where $\vec{x}$ is the measured signal, $\vec{d}$ is the feature impulse signal, and $\vec{e}$ is the noise signal. These components represent important characteristic information or responses under fault situations in pipelines. The MED filter can take advantage of the maximization kurtosis to identify a solution for filtering noise. The iterative selection is derived by solving for $\vec{f\;}$ in the Wiggen’s method, and the kurtosis maximization problem is described as

(2) $$\underset{\vec{\;f}}{\max\;}kurtosis=\underset{\;\vec{f}}{\max}\frac{\sum_{n=1}^{N}y_{n}^{4}}{{\left(\sum_{n=1}^{N}y_{n}^{2}\right)}^{2}},\;\vec{y}=\left[\begin{array}{c} y_{1}\\ y_{2}\\ \begin{array}{c} \vdots \\ y_{n} \end{array} \end{array}\right],\vec{\;f}=\left[\begin{array}{c} f_{1}\\ f_{2}\\ \begin{array}{c} \vdots \\ f_{n} \end{array} \end{array}\right],$$

The output $\vec{y}$ can be calculated as

(3) $$\vec{y}=\vec{f}*\vec{x},\vec{y\;}=\sum_{l=1}^{L}f_{1}x_{k-l+1},\;k=1,2,\cdots ,N\;$$

Kurtosis was applied to evaluate multiple components and calculate the peak for the $\vec{d\;}$ component because it is large for the fault impulse component and small for the noise component. Therefore, for comparison with other signal components, a filter $\vec{f}$ is designed to maximize the kurtosis parameter to remove the noise signal and obtain the approximate fault feature signal with a high kurtosis to exact feature impulse signals from the measured signal.

(4) $$\vec{y}={\bar{\;X}}_{0}^{T}\vec{f\;}$$

(5) $${\bar{\;X}}_{0}=\left(\begin{array}{ccccc} x_{1} & x_{2} & x_{3} & & x_{N}\\ & x_{1} & x_{2} & \cdots & x_{N-1}\\ & & x_{1} & & x_{N-2}\\ & \vdots & & \ddots & \vdots \\ 0 & 0 & 0 & \cdots & x_{N-L+1} \end{array}\right),\;\vec{\;f\;}=\frac{\sum_{n=1}^{N}y_{n}^{2}}{\sum_{n=1}^{N}y_{n}^{4}}{\left({\bar{X}}_{0}{\bar{X}}_{0}{}^{T}\right)}^{-1}{\bar{X}}_{0}\left[y_{1}^{3}y_{2}^{3}\cdots y_{N}^{3}\right]$$

The initial difference filter from $\vec{f}=\left[0,\cdots ,0,1,-1,0,\cdots ,0\right]$ was used to calculate the filter $\vec{f}$ to calculate output $\vec{y\;}$ as an important feature impulse signal.

**Figure 1:**
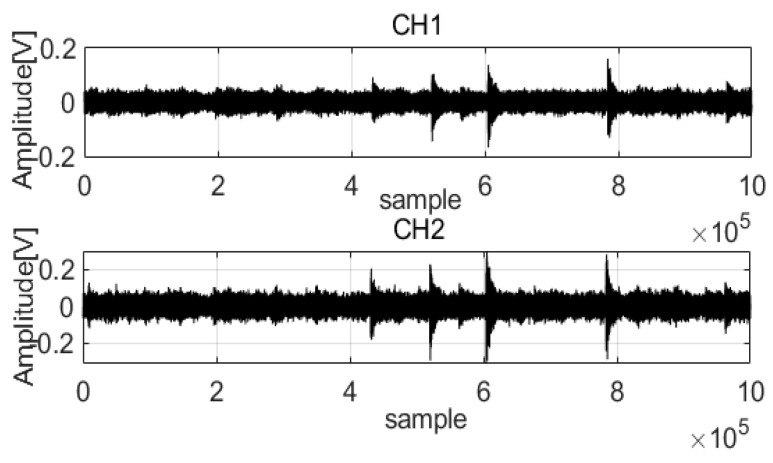
Acoustic waves of the two channels.

Acoustic wave data from pipelines were used for analyses to verify the performance of the MED method (data parameters: leak size 2 mm, pressure 18 bar). Figure 1 shows that the acoustic waves acquired correspond to a 2 mm leak size and 18 water bar pressure from two channels (CH1 and CH2). There is noise in the acoustic waves that mixes with the feature signals, which is a disadvantage of analysis. A length equal to 1,000,000 data points was considered to extract the feature signals.

**Figure 2:**
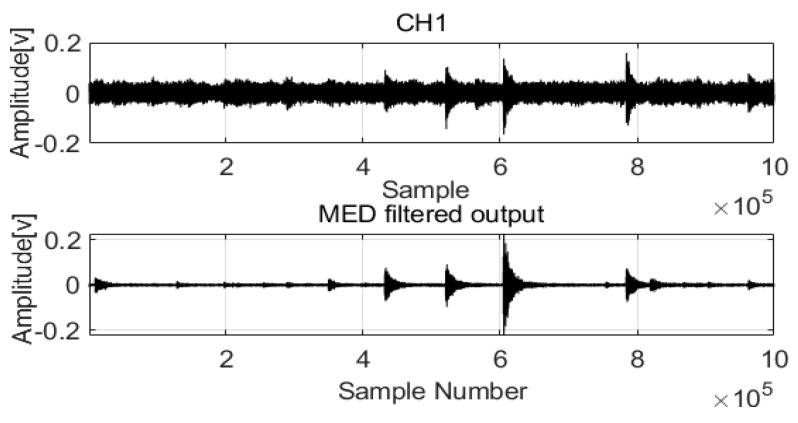
MED method for the acoustic signals in Channel 1.

**Figure 3:**
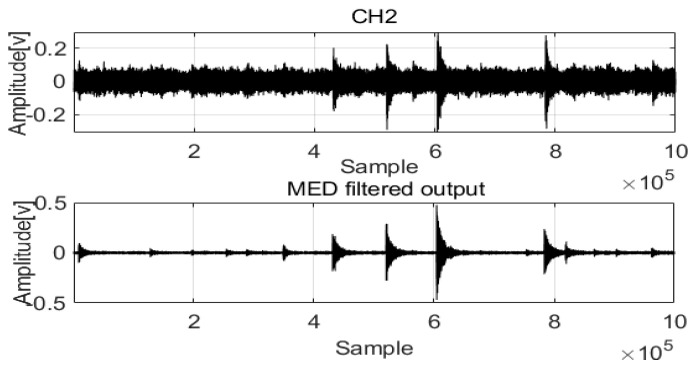
MED method for the acoustic signals in Channel 2.

The raw signals were processed by MED to remove noise. The two original signals are compared in Figure 2 and Figure 3.

The acoustic signals of Channel 1 and Channel 2 installed at two positions in the pipeline are saved with the noise and feature impulse data. MED successfully used a maximization kurtosis norm in filtering to remove noise from the mixed signals. After MED processing, the feature impulse signals are easily detected for damping frequency energy processing.

## 3. The Time Difference of Acoustic Waves

A new approach based on damping frequency energy in leak displacement is designed to identify the arrival time by transforming the signal into a response domain of this damping term into the dynamic differential equation and tracking the damping frequency energy. This can take advantage of a correspondingly high frequency to reflect the trend of the feature signals. The damping frequency energy is designed to extract important energy information and yields a smooth envelope over time for the feature signals. Intersection with zero can track the first arrival time through the envelope changes and detect the time difference for acoustic waves in two channels. The dynamic differential equation with damping term is described as

(6) $$M\ddot{U}+C\dot{U}+KU=F\;$$

where M is the similar mass, *C* is the damping term, K is the stiffness term, $F=-M\ddot{U_{g}}$, and $U_{t}=U+U_{g}$, where U is the relative displacement, and $U_{t}$ is the absolute place of similar mass. Equation (6) can be derived to Equation (7):

(7) $$\frac{\;M{\left[\dot{U_{g}}+\dot{U}\right]}^{2}}{2}+\int C\dot{U}dU+\int KUdU=\int M\left[\ddot{U_{g}}+\ddot{U}\right]dU_{g}$$

where $U_{g}$ is the ground displacement. Integrating the above equations with respect to U gives the absolute energy formulation, or the right side of Equation (7) can be rewritten as:

(8) $$\int M\left[\ddot{U_{g}}+\ddot{U}\right]dU_{g}=\int_{0}^{t}M\left[\ddot{U_{g}}+\ddot{U}\right]\dot{U_{g}}dt\;$$

where *t* is time. Equation (8) can describe the energy as follows:

(9) $$E_{k}+E_{\zeta }+E_{s}=E_{I}\;$$

where $E_{K}$ is the absolute kinetic energy, $E_{\zeta }$ is the damping frequency energy, $E_{s}$ is the elastic strain energy, and $E_{I}$ is the absolute input energy. From Equation (9), $E_{\zeta }$ is derived as

(10) $$E_{\zeta }=\int 2\zeta \omega_{D}{\dot{U}}^{2}dt\;$$

where $\omega_{D}=2\pi T_{D}/\sqrt{1-\zeta^{2}}$ is the cyclic frequency, and $T_{D}$ is the damping.

**Figure 4:**
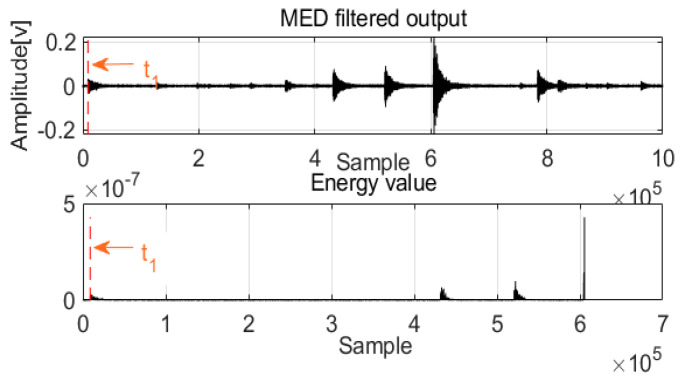
The first arrival time for Channel 1.

**Figure 5:**
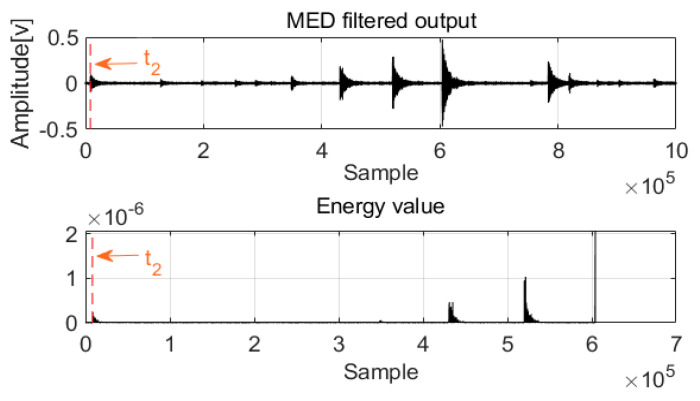
The first arrival time for Channel 2.

After MED processing of acoustics signals in two channels, a new method was designed to detect the first arrival time. For damping frequency energy, the damping ratio is typically selected as impulse waves; at this damping energy level, the frequency response approaches the Butterworth maximally flat magnitude filter. With these special frequencies and damping energy, the architecture can achieve equilibrium quickly, and the relative motion of the mass is extremely small, which is an advantage of the system. The damping energy with frequency is a cumulative function that is proportional to the square of the relative velocity, and it can produce a smooth envelope over time. Before the first arrival of the acoustic waves, the frequency energy valve is zero or near zero, and the first arrival time can be detected as shown in Figure 4 and Figure 5.

**Figure 6:**
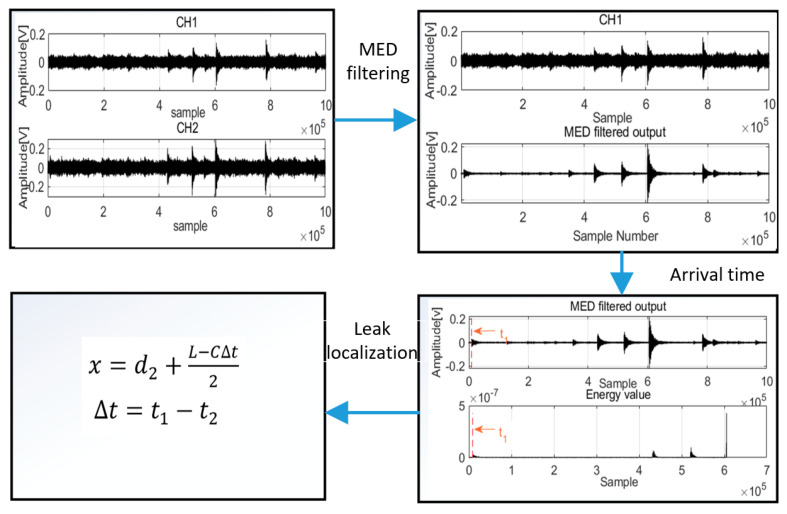
The process of the proposed method architecture.

When the difference of the first arrival time of the two channels is combined with the velocity value, the leak can be localized. This damping frequency energy is very sensitive to noise; as a result, the noise is removed from the acoustic signals by MED, which is advantageous for zero crossing detection. This architecture uses the smoothness attribute of damping frequency energy to detect the zero crossing and ensure the first arrival time of the acoustic signals using the AIC method. This part depicts a method based on frequency energy to detect the first arrival time for AE signals and localizes the leak according to

(11) $$x=d_{2}+\frac{L-C\Delta t}{2}\;$$

(12) $$\Delta t=t_{1}-t_{2}\;$$

where *c* is the velocity, *L* is the distance between Sensor 1 and Sensor 2, $t_{1}\;$ is the first arrival time for acoustic waves in Channel 1, $t_{2}$ is the first arrival time for acoustic waves in Channel 2, and *x* is the leak location. The whole process of the architecture is shown in Figure 6.

Implemented procedure:

(1) Two AE signals are acquired from the pipeline for filter processing. In the first filter process, MED is used to remove noise from mixed signals.
(2) After filter processing, the first arrival time t is detected from denoising signals by the new method based on damping frequency energy. Before time t, the energy value is zero or near zero; however, at time t, the energy value is greater than zero, and it is the time that we want to get for acoustic waves.
(3) Finally, the leak is localized using Equation (11). This allows for enactment of measures to be initiated to protect the pipelines.

## 4. Experiment

### 4.1. Experiment Setup

**Figure 7:**
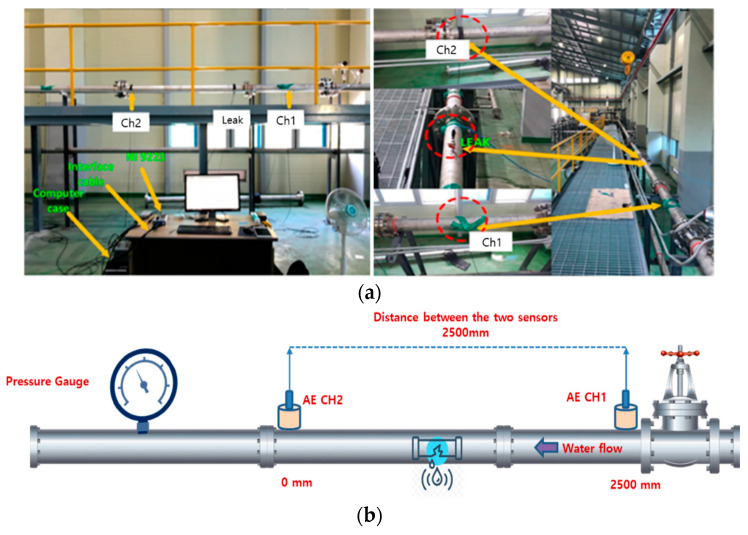
Experimental setup used for simulating leaks in the pipeline: (**a**) photo and (**b**) schematic.

To validate the performance of the proposed model, the experiment based on AE signals for pipelines is shown in Figure 7, including the complete details of the experimental setup: (a) photos and (b) schematic. The experimental setup consists of a water pipeline made of stainless steel with an outer diameter of 114 mm and a thickness of 6 mm. In addition, the AE equipment system is installed on a water pipeline, and R151-AST sensors from the MITRAS Corporation are used to collect AE data. These sensors’ specification is summarized in Table 1. A 16-bit analog-to-digital converter with controllable sampling frequency and interface module via high-speed universal serial bus standard is integrated in an NI-9223 module manufactured by the National Instruments company. In the experiment, a leak valve is connected to a hose for transporting the fluid into a container. The experimental information is shown in Table 2.

Initially, the leak valve is closed, and the water pipeline is operated at normal conditions. When the water pressure and leak valve size are changed, different types of signals are produced. Finally, the pipeline’s sensor data are gathered for different leak sizes of 0.3 mm, 0.5 mm, 1 mm, and 2 mm at the different pressure levels including 7, 13, and 18 bars. Four leaks with the diameter of 0.3, 0.5, 1.0, and 2.0 mm are represented by $F=\{F_{1},\;F_{2},\;F_{3},\;F_{4}\}$, respectively, and three types of the pressure level of 13, 18, and 7 bars are represented by $P=\{P_{1},\;P_{2},\;P_{3}\}$. In the experiment, for a particular leak size and pressure level, each signal sample is collected and considered for further analysis.

### 4.2. Results and Discussion

**Figure 8:**
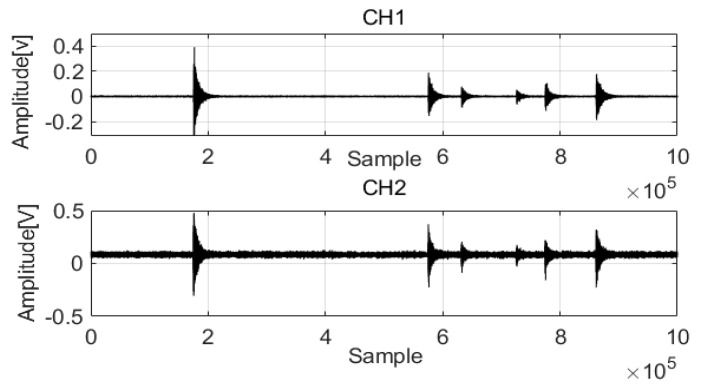
Acoustic waves of two channels.

To verify the performance of the proposed method, many acoustic wave data from pipelines were used in the analysis (data parameters: leak size 0.5 mm, pressure 18 bar). Figure 8 shows the acoustic wave acquired from two channels (CH1 and CH2). Noise was present in the acoustic waves and was mixed with the feature signals, complicating analysis. To extract the feature signals, a length equal to 1,000,000 data points was considered.

**Figure 9:**
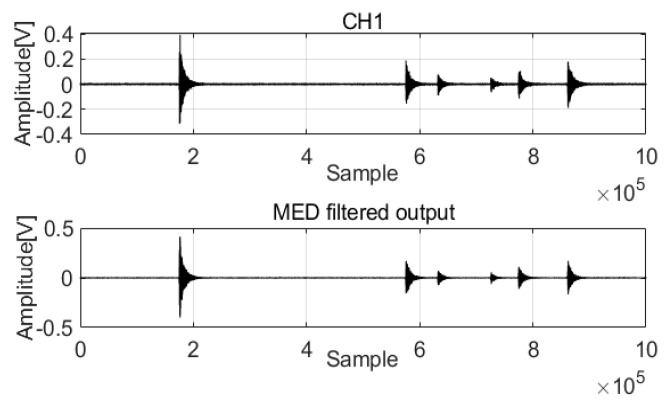
MED method for the acoustic signals in Channel 1.

**Figure 10:**
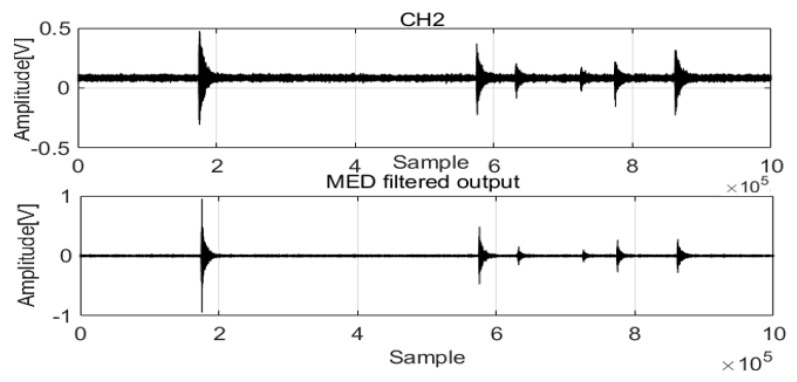
MED method for the acoustic signals in Channel 2.

The original signals were processed by MED to remove noise, and the two filtered signals were compared. The results are shown in Figure 9 and Figure 10.

**Figure 11:**
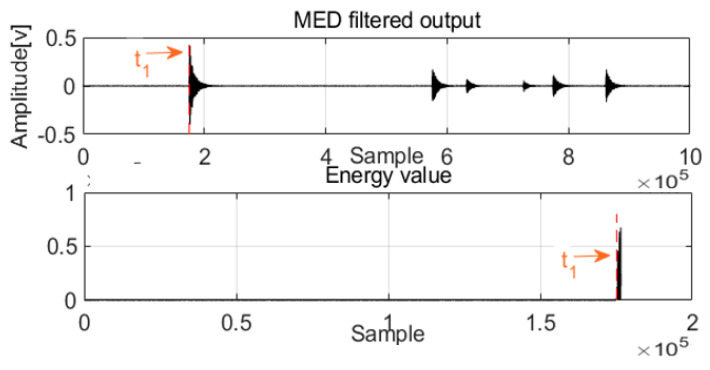
The first arrival time for Channel 1.

**Figure 12:**
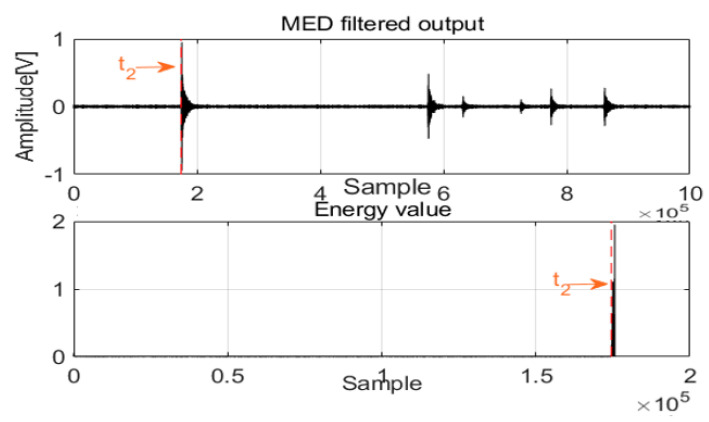
The first arrival time for Channel 2.

As with acoustic signals such as those in Channel 1 and Channel 2, MED can use the maximization kurtosis norm to remove noise from the mixed signals. After MED processing, the feature impulse signals were detected for easy damping frequency energy processing. To validate leak localization in the pipelines, Figure 11 and Figure 12 show the first arrival times for Channel 1 and Channel 2. Combined with the velocity to localize the leak, the result is 859 mm, and the relative error is 1.64%. To validate the performance of the proposed method compared with those of the GCC and EMD + GCC methods, the correct leak localization for the GCC method was 1086 mm, and the relative error was 7.44%. For the EMD + GCC method, the leak localization result was 1027 mm, and the relative error was 5.08%. These findings demonstrate better leak localization with the proposed method than with the others.

**Figure 13:**
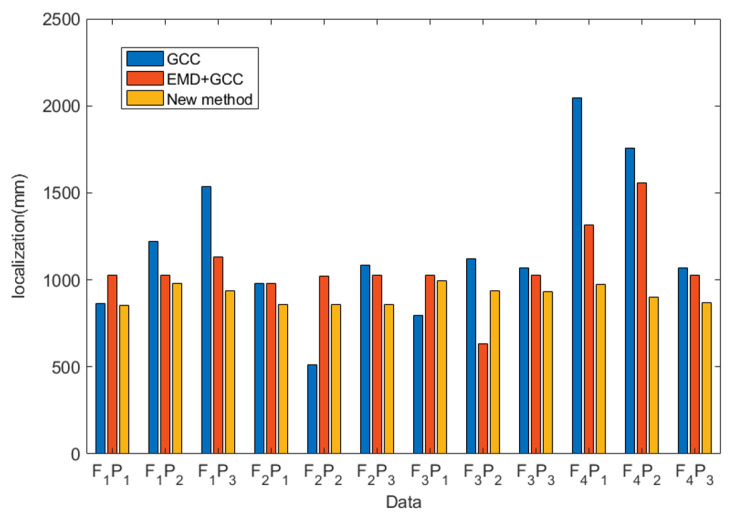
Leak localization with the tested methods.

**Figure 14:**
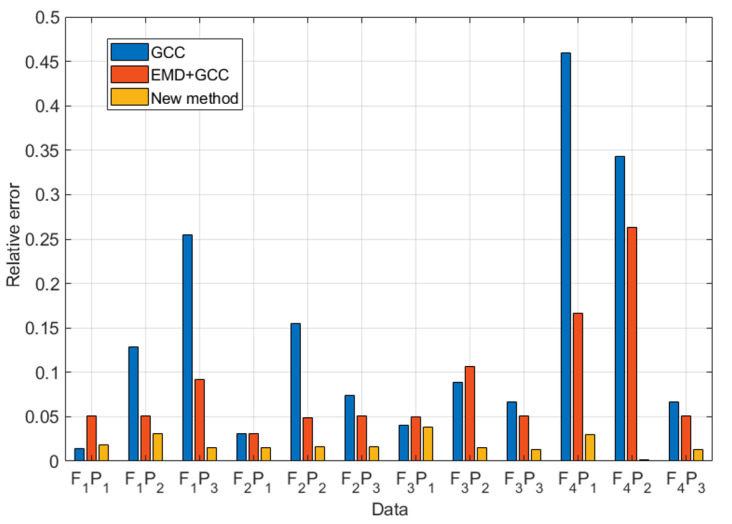
Relative error of the tested methods.

To further validate the performance of the proposed method in industrial pipelines, data were applied to compare the GCC and EMD + GCC methods with the proposed method. The computational result is shown in Figure 13 and Figure 14.

Data $F_{1}P_{1}$: The GCC method can achieve 865 mm for leak localization with a relative error of 1.4%. The result of EMD + GCC was 1027 mm, and the relative error was 5.08%. That of the proposed method was 854 mm, and the relative error was 1.84%.

Data $\;F_{1}P_{2}$: The GCC method produced a finding of 1222 mm, and the relative error was 12.88%. The EMD + GCC method was 1027 mm, and the relative error was 5.08%. The proposed method was 977 nm, and the relative error was 3.08%.

Data $F_{1}P_{3}$: The GCC method localized the leak at 1536 mm, and the relative error was 25.44%. The EMD + GCC finding was 1131 mm, and the relative error was 9.24%. The proposed method localized the leak at 939 mm, and the relative error was 1.56%.

Data $\;F_{2}P_{1}$: The GCC method can achieve 978 mm, and the relative error was 3.12%. The EMD + GCC method was 978 mm, and the relative error was 3.12%. The proposed method was 863 mm, and the relative error was 1.56%.

Data $F_{2}P_{2}$: The GCC method can achieve 512 mm for leak localization, and the relative error was 15.52%. The EMD + GCC was 1023 mm, and the relative error was 4.92%. The proposed method was 858 mm, and the relative error was 1.68%.

Data $\;F_{2}P_{3}$: The GCC method can achieve 1086 mm, and the relative error was 7.44%. The EMD + GCC method was 1027 mm, and the relative error was 5.08%. The proposed method was 859 mm, and the relative error was 1.64%.

Data $F_{3}P_{1}$: The GCC method can achieve 798 mm for leak localization, and the relative error was 4.08%. The EMD + GCC was 1026 mm, and the relative error was 5.04%. The proposed method was 997 mm, and the relative error was 3.88%.

Data $\;F_{3}P_{2}$: The GCC method can achieve 1122 mm, and the relative error was 8.88%. The EMD + GCC method was 633 mm, and the relative error was 10.68%. The proposed method was 938 mm, and the relative error was 1.52%.

Data $F_{3}P_{3}$: The GCC method can achieve 1067 mm for leak localization, and the relative error was 6.68%. The EMD + GCC was 1027 mm, and the relative error was 5.08%. The proposed method was 932 mm, and the relative error was 1.28%.

Data $\;F_{4}P_{1}$: The GCC method can achieve 2048 mm, and the relative error was 45.92%. The EMD + GCC method was 1316 mm, and the relative error was 16.64%. The proposed method was 974 mm, and the relative error was 2.96%.

Data $F_{4}P_{2}$: The GCC method can achieve 1758 mm for leak localization, and the relative error was 34.32%. The EMD + GCC was 1559 mm, and the relative error was 26.36%. The proposed method was 903 mm, and the relative error was 0.12%.

Data $\;F_{4}P_{3}$: The GCC method can achieve 1067 mm, and the relative error was 6.68%. The EMD + GCC method was 1027 mm, and the relative error was 5.08%. The proposed method was 867 mm, and the relative error was 1.32%.

To further verify the effectiveness of the GCC, EMD + GCC, and proposed methods in industrial pipelines, the results are shown in Table 3 and Table 4.

According to the analysis of the above comparison, the GCC method can detect a leak position in pipelines based on the TDOA. However, there are various environmental and internal factors that influence the quality of GCC, such as noise, so the GCC was combined with EMD filtering to increase the prediction accuracy. However, a further increase in quality is needed, so the new hybrid method was proposed to filter noise and detect the first arrival time for more accurate pipeline leak localization.

## 5. Conclusions

In this paper, a novel hybrid approach based on the MED filter method and damping frequency energy was designed to remove the noise from acoustic waves and achieve leak localization in industrial pipelines. The novel approach has the following advantages:

(1) Two sensors are installed at each end of the industrial pipeline to collect AE signals from each channel. Collected AE signals include environment noises, which prevents intelligent analysis of leak localization. To address this issue, MED is used with the maximization kurtosis norm of acoustic signals to remove the noise and extract informative feature signals.
(2) The damping frequency energy based on the dynamic differential equation with damping term was designed to extract important energy information with frequency, and a smooth envelope over time for feature signals was then produced. Zero crossing can track the arrival time through envelope changes and detect the time difference of AE waves from two channels, combining them with velocity to localize the leak. Compared with existing methods, the proposed approach provides better leak localization over the conventional GCC and EMD-GCC methods.
(3) As industrial pipelines operate in various environment noises and are influenced by internal factors, intelligent analysis of leak localization is required. To address these issues, we will consider additional parameters in the proposed method and perform more experiments for the accurate leak localization.

## Figures and Tables

**Table 1:** Operating specification of R151-AST.

No	Parameter	Value
1	Peak sensitivity, ref [V/(m/s)]	109 [dB]
2	Peak sensitivity, ref [V/μbar]	−22 [dB]
3	Operating frequency range	50–400 [kHz]
4	Resonant frequency, ref [V/(m/s)]	75 [kHz]
5	Resonant frequency, ref [V/μbar]	150 [kHz]
6	Directionality	±1.5 [db]
7	Temperature range	−35 to 70 [°C]

**Table 2:** Pipeline experiment information.

No	Quantity	Detail
1	Location of Sensor 1 (d1)	2600 [mm]
2	Location of Sensor 2 (d2)	100 [mm]
3	Location of leak (d)	900 [mm]
4	Thickness of pipelines	6.02 [mm]
5	Outer diameter of pipelines	114.3 [mm]
6	Material of pipelines	Stainless steel 304
7	Wave velocity (C)	1,500,000 [mm/s]

**Table 3:** Leak localization with the tested methods.

Data	GCC [mm]	GCC + EMD [mm]	Proposed Method [mm]
F1P1	865	1027	854
F1P2	1222	1027	977
F1P3	1536	1131	939
F2P1	978	978	861
F2P2	512	1023	858
F2P3	1086	1027	859
F3P1	798	1026	997
F3P2	1122	633	938
F3P3	1067	1027	932
F4P1	2048	1316	974
F4P2	1758	1559	903
F4P3	1067	1027	867

**Table 4:** Relative error of the tested methods.

Data	GCC [%]	GCC + EMD [%]	Proposed Method [%]
F1P1	1.4	5.08	1.84
F1P2	12.88	5.08	3.08
F1P3	25.44	9.24	1.56
F2P1	3.12	3.12	1.56
F2P2	15.52	4.92	1.68
F2P3	7.44	5.08	1.64
F3P1	4.08	5.04	3.88
F3P2	8.88	10.68	1.52
F3P3	6.68	5.08	1.28
F4P1	45.92	16.64	2.96
F4P2	34.32	26.36	0.12
F4P3	6.68	5.08	1.32

## Data Availability

The data are from the industry. Due to the privacy policy of the industry, the data are not available publicly.
